# Development and validation of a nomogram for predicting treatment failure in culture-negative peritoneal dialysis–associated peritonitis

**DOI:** 10.1093/ckj/sfaf390

**Published:** 2025-12-13

**Authors:** Lingling Niu, Pan Dou, Yanyan Wang, Feng Li, Dandan Zhang, Jing Li, Xiaofen Ma, Chengjuan Fan, Xiang Li, Yiming Zhang

**Affiliations:** Department of Gastroenterology, Affiliated Hospital of Jining Medical University, Jining, China; Department of Nephrology, Affiliated Hospital of Jining Medical University, Jining, China; Department of Nephrology, Affiliated Hospital of Jining Medical University, Jining, China; Department of Nephrology, Zaozhuang Municipal Hospital, Zaozhuang, China; Department of Nephrology, Heze Municipal Hospital, Heze, China; Department of Nephrology, Zaozhuang Municipal Hospital, Zaozhuang, China; Department of Nephrology, Affiliated Hospital of Jining Medical University, Jining, China; Department of Nephrology, Affiliated Hospital of Jining Medical University, Jining, China; Department of Nephrology, Affiliated Hospital of Jining Medical University, Jining, China; Department of Nephrology, Affiliated Hospital of Jining Medical University, Jining, China

**Keywords:** CNPDP, nomogram, online tool, risk prediction, treatment failure

## Abstract

**Background:**

Culture-negative peritoneal dialysis–associated peritonitis (CNPDP) carries a high risk of treatment failure but lacks validated prediction tools. This study aimed to develop and validate a clinical nomogram for individualized risk assessment of treatment failure in CNPDP patients.

**Methods:**

In this multicenter retrospective study, 288 CNPDP patients treated at Jining Medical University Affiliated Hospital (2013–23) were randomly allocated to training (*n* = 173) and internal validation (*n* = 115) cohorts. An independent external cohort (*n* = 103) from Zaozhuang Municipal Hospital and Heze Municipal Hospital assessed generalizability. First, we used Random Forest to estimate missing data for variables with <30% missing values. Then, we used LASSO regression to analyze 32 candidate predictors. These predictors covered areas like patient demographics, clinical scores and lab test results. The final multivariate logistic regression model was visualized as a clinical nomogram. Performance was rigorously evaluated through area under receiver operating characteristic curve (AUC), calibration plots and decision curve analysis. The primary endpoint was composite treatment failure (catheter removal or peritonitis-related mortality ≤30 days).

**Results:**

LASSO identified five independent predictors: effluent white blood cell count on Day 3 (Eff_WBC_D3), serum albumin (ALB), total cholesterol (TC), magnesium (Mg) and phosphorus (P). The nomogram achieved excellent discrimination: training cohort AUC = 0.897 (95% confidence interval 0.817–0.978), internal validation AUC = 0.861 (0.770–0.952) and external validation AUC = 0.849 (0.750–0.948) with minimal optimism (ΔAUC = 0.036). Eff_WBC_D3 demonstrated the strongest univariate predictive power (AUC = 0.830). Calibration curves showed optimal fit (Hosmer–Lemeshow *P *= .32), while decision curve analysis confirmed clinical utility across probability thresholds of 5%–50%. For bedside implementation, an interactive web tool was developed (https://liuliangmianhua.shinyapps.io/dynnomapp/).

**Conclusion:**

This externally validated five-variable nomogram, deployed as a freely accessible online tool, offers a robust, practical tool for predicting treatment failure in CNPDP. Its integration of dynamic dialysate markers with routine laboratory data enables personalized early intervention and supports timely clinical decision-making.

KEY LEARNING POINTS **What was known:**

- Culture-negative peritoneal dialysis–associated peritonitis (CNPDP) is common and carries a high risk of treatment failure, but validated prediction tools are lacking.
- Existing prognostic models for PD-associated peritonitis are primarily derived from and targeted at culture-positive episodes.
- The distinct clinical course of CNPDP necessitates specific risk stratification tools for early intervention.

**This study adds:**

- We developed and validated a novel nomogram incorporating five variables (Eff_WBC_D3, ALB, TC, Mg, P) to predict treatment failure in CNPDP.
- The model highlights the prognostic importance of persistent inflammation (Eff_WBC_D3), nutritional status (ALB, TC) and mineral metabolism (Mg, P).
- An interactive online tool was created to facilitate real-time, bedside risk calculation (https://liuliangmianhua.shinyapps.io/dynnomapp/).

**Potential impact:**

- This tool enables personalized early risk assessment and supports timely clinical decision-making for CNPDP patients.
- It has the potential to optimize resource allocation by identifying high-risk patients requiring intensified monitoring or intervention.
- The online platform promotes easy integration of the model into routine clinical workflow.

## INTRODUCTION

Peritoneal dialysis (PD) is a widely used renal replacement therapy for patients with end-stage renal disease (ESRD), offering advantages such as better preservation of residual renal function and greater patient autonomy compared with hemodialysis [1, 2]. However, PA-associated peritonitis (PDAP) remains a major complication, contributing significantly to technique failure, hospitalization and mortality [3–6]. Notably, 13.4%–40% of PDAP episodes are culture-negative, a variability which may be influenced by differences in culture techniques, prior antibiotic use and local microbiological flora [7–9]. Their undefined etiology complicates antimicrobial treatment, often resulting in prolonged therapy and increased risks of catheter loss and death. Existing prognostic models for PDAP primarily focus on culture-positive cases [10–12], largely overlooking the distinct clinical features and pathophysiology of culture-negative PDAP (CNPDP). Models derived from culture-positive cohorts are ill-suited for CNPDP, as its clinical course is driven not by specific microbial virulence but predominantly by host inflammatory and nutritional responses, in the absence of pathogen-directed therapy.

Therefore, identifying reliable predictors of treatment failure in CNPDP is essential for effective risk stratification and personalized management. This study aimed to develop a clinical prediction model for CNPDP treatment failure using logistic regression and nomogram construction, which was subsequently deployed as an interactive web-based tool. The proposed model may improve early risk assessment, inform timely therapeutic decisions and ultimately help optimize outcomes in this high-risk patient population.

## MATERIALS AND METHODS

### Patient population

This study employed a retrospective cohort design comprising two distinct phases. First, the derivation phase: single-center model development at Jining Medical University Affiliated Hospital (October 2013–October 2023). Second, the validation phase: external validation using independently retrospective cohorts from Zaozhuang Municipal Hospital and Heze Municipal Hospital. To ensure generalizability, all participating centers adhered to the 2022 International Society for Peritoneal Dialysis (ISPD) guidelines for diagnosing CNPDP. Standardized protocols were uniformly implemented for microbiological culture, laboratory analysis and data extraction with dual verification by nephrologists. This guaranteed consistent data quality across sites for model development and validation. All patients were followed until the occurrence of treatment failure or for a minimum of 30 days post-diagnosis, ensuring complete endpoint ascertainment.

Inclusion criteria:

(i) Age ≥18 years;
(ii) Diagnosis of ESRD maintained on continuous ambulatory PD for >3 months;
(iii) CNPDP diagnosis confirmed according to the 2022 ISPD guidelines [13], requiring both: PD effluent white blood cell (WBC) count >100/μL with ≥50% polymorphonuclear neutrophils; negative aerobic and anaerobic cultures after standard 5-day incubation;
(iv) Complete baseline clinical and laboratory data.

Exclusion criteria:

(i) Delay of formal treatment for more than 72 h;
(ii) Active non-peritonitis infections at enrollment;
(iii) Peritonitis episodes within 90 days before recruitment;

(iv) Incomplete follow-up documentation or loss to follow-up;
(v) Renal transplantation during active peritonitis therapy;
(vi) Life-limiting comorbidities including but not limited to metastatic cancer, severe decompensated heart failure or terminal liver disease, as defined by the attending physician’s assessment that the condition itself could independently affect the primary outcome.

A flowchart detailing the patient selection process is provided in the Supplementary data, Fig. S1.

This study was conducted in accordance with the Declaration of Helsinki and reported following the Strengthening the Reporting of Observational Studies in Epidemiology (STROBE) guidelines. It was approved by the Ethics Committee of the Affiliated Hospital of Jining Medical University (Approval No.: 2023–10-C032), and the requirement for informed consent was waived due to its retrospective design and use of anonymized data. All data from both the primary and external validation centers (Zaozhuang and Heze) were de-identified prior to analysis to ensure confidentiality.

### Study parameters and endpoint

Demographic and laboratory parameters were retrieved from electronic medical records. Data from all three hospitals were extracted using Structured Query Language (SQL) queries followed by mandatory dual verification by independent nephrologists, ensuring data integrity and reliability through this standardized protocol. Discrepancies (<6% of items) were resolved by panel review. Collected variables included:

• demographics: sex, age (years), body mass index (BMI);
• clinical scores: Numerical Rating Scale for pain (pain NRS) [14] and Nutritional Risk Screening 2002 (NRS 2002) [15];
• serial peritoneal dialysate effluent WBC counts (/μL) were measured at the following timepoints: pre-treatment (Eff_WBC_Pre) and on Days 1–4 after initiation of treatment (Eff_WBC_D1, Eff_WBC_D2, Eff_WBC_D3, Eff_WBC_D4).
• hematological parameters: WBC count (WBC, ×10^9^/L), hemoglobin (HGB, g/L), mean corpuscular volume (MCV, fL), neutrophil count (NEUT, ×10^9^/L);
• inflammatory markers: C-reactive protein (CRP, mg/L), procalcitonin (PCT, μg/L);
• biochemical indices: serum albumin (ALB, g/L), glucose (GLU, mmol/L), triglycerides (TG, mmol/L), total cholesterol (TC, mmol/L), low-density lipoprotein cholesterol (LDL-C, mmol/L), high-density lipoprotein cholesterol (HDL-C, mmol/L), creatinine (Cr, μmol/L), urea (mmol/L), uric acid (UA, μmol/L);
• electrolytes: serum potassium (K, mmol/L), serum bicarbonate (HCO_3_⁻, mmol/L), serum calcium (Ca, mmol/L), serum magnesium (Mg, mmol/L), serum phosphate (P, mmol/L);
• hormonal markers: parathyroid hormone (PTH, pg/mL), b-type natriuretic peptide (BNP, pg/mL);

The primary endpoint was treatment failure, defined as peritoneal catheter removal or all-cause mortality directly related to peritonitis within 30 days after diagnosis of CNPDP [13].

### Statistical analysis

Baseline characteristics were compared across the training, internal validation and external validation cohorts. Categorical variables were summarized as frequencies and percentages, and compared using the Chi-square test with standardized residuals analysis, or the Fisher–Freeman–Halton test when expected cell counts were small. Continuous variables were presented as mean ± standard deviation if normally distributed, and as median with interquartile range otherwise. Normality was assessed using the Shapiro–Wilk test. Non-normally distributed continuous variables were compared using the Kruskal–Wallis test, followed by Dunn’s post-hoc pairwise comparisons with appropriate adjustments for multiple testing.

Missing data with <30% missingness were imputed using the Random Forest–based missForest algorithm (R package missForest v1.5), while variables with >30% missingness were excluded from further analysis.

In the training cohort, candidate predictors were first screened using 10-fold cross-validated least absolute shrinkage and selection operator (LASSO) logistic regression. Variables with no evidence of multicollinearity (variance inflation factor <5) were then entered into a multivariable logistic regression model to construct the final predictive model.

Model discrimination was assessed using receiver operating characteristic (ROC) curves and quantified by the area under the curve (AUC). Calibration was evaluated with calibration plots, and clinical utility was examined using decision curve analysis (DCA) to estimate net clinical benefit across a range of threshold probabilities.

All statistical tests were two-sided, with a significance threshold of *P* < .05. Analyses were conducted in R version 4.2.2 (packages: rms, pROC, rmda) and MSTATA version 3.0.

The sample size for model development was assessed based on the events per variable (EPV) principle. For the five predictors in the final model, the training cohort (*n* = 173) with 22 treatment failure events yielded an EPV of 4.4. This is close to the commonly accepted minimum threshold of EPV ≥5 for logistic regression models, and the robust validation performance subsequently confirmed the model’s stability.

The final nomogram was deployed as an interactive, web-based application using the R Shiny framework (hosted on shinyapps.io) to enable real-time, point-of-care risk estimation.

## RESULTS

### Baseline characteristics

The study population comprised 462 initially screened CNPDP patients from three tertiary centers between 2013 and 2023. After applying uniform exclusion criteria (detailed in Supplementary data, Fig. S1), 355 patients qualified for analysis: the development cohort (Jining Center, *n* = 288) was partitioned into a training cohort (*n* = 173) for model construction and an internal validation cohort (*n* = 115) for preliminary testing, while an independent external validation cohort (Zaozhuang/Heze centers, *n* = 103) was used for final model evaluation.

The baseline demographic and clinical characteristics of the study cohorts are summarized in detail. Gender distribution was similar across all cohorts, with males accounting for 60.1% in the training cohort, 55.7% in the internal validation cohort and 55.3% in the external validation cohort (*P* = .657). Age distributions were also comparable, with mean ages of 46.0 ± 13.2 years, 47.9 ± 13.3 years and 47.4 ± 13.8 years, respectively (*P* = .485). BMI did not differ significantly among cohorts, with means ranging from 23.7 ± 4.6 to 24.2 ± 4.6 kg/m^2^ (*P* = .503). All cohorts demonstrated comparable baseline characteristics with no statistically significant differences (all *P* > .05; Table 1), confirming appropriate stratification for model development. Treatment failure rates in CNPDP patients were also comparable across cohorts: 22 cases (12.7%) in the training cohort, 14 cases (12.2%) in the internal validation cohort and 15 cases (14.6%) in the external validation cohort, with no statistically significant difference (*P* > 0.05).

**Table 1:** Baseline characteristics of the training, internal validation and external validation cohorts.

	Cohort			
Characteristic	Training cohort, *N* = 173	Internal test cohort, *N* = 115	External test cohort, *N* = 103	*P*
Male, *n* (%)	104 (60.1)	64 (55.7)	57 (55.3)	.657
Age (years)	46.0 ± 13.22	47.9 ± 13.27	47.4 ± 13.79	.485
BMI (kg/m^2^)	23.7 ± 4.58	24.2 ± 4.57	24.1 ± 4.39	.503
Pain NRS	1.0 (0.0, 2.0)	1.0 (0.0, 2.0)	1.0 (0.0, 2.0)	.419
NRS 2002	1.0 (1.0, 2.0)	1.0 (1.0, 1.4)	1.0 (1.0, 2.0)	.381
Eff_WBC_Pre (/µL)	1200.0 (320.0, 3759.5)	1203.0 (279.0, 2720.0)	1247.0 (340.0, 4543.0)	.539
Eff_WBC_D1 (/µL)	454.0 (170.0, 1194.0)	384.3 (118.0, 1567.0)	667.8 (176.0, 2100.0)	.169
Eff_WBC_D2 (/µL)	112.0 (40.0, 391.2)	128.0 (36.0, 473.6)	189.3 (61.0, 849.0)	.076
Eff_WBC_D3 (/µL)	72.4 (23.0, 192.7)	60.0 (18.0, 220.0)	114.4 (36.0, 338.0)	.064
Eff_WBC_D4 (/µL)	43.1 (15.7, 136.6)	42.0 (13.0, 131.0)	53.0 (15.7, 208.6)	.657
WBC (×10^9^/L)	8.0 ± 3.03	8.2 ± 3.58	8.1 ± 3.45	.998
HGB (g/L)	94.6 ± 22.48	98.4 ± 22.18	96.0 ± 22.59	.343
MCV (fL)	91.6 ± 5.94	91.3 ± 5.62	91.3 ± 5.93	.800
NEUT(×10^9^/L)	5.6 (4.2, 7.6)	5.9 (4.0, 7.9)	6.2 (4.3, 8.2)	.517
CRP (mg/L)	26.7 (7.9, 64.4)	30.8 (6.1, 85.9)	25.3 (7.8, 82.0)	.711
PCT (μg/L)	1.3 (0.5, 7.1)	1.6 (0.6, 5.7)	1.5 (0.6, 7.1)	.849
ALB (g/L)	30.4 ± 5.89	30.6 ± 5.57	33.1 ± 5.83	.828
GLU (mmol/L)	5.2 (4.5, 6.3)	5.1 (4.4, 6.2)	5.1 (4.4, 5.9)	.506
TG (mmol/L)	1.1 (0.7, 1.5)	1.1 (0.8, 1.4)	1.2 (0.9, 1.7)	.312
TC (mmol/L)	3.8 (3.4, 4.4)	3.9 (3.3, 4.5)	3.9 (3.2, 4.5)	.821
LDL-C (mmol/L)	2.1 (1.8, 2.5)	2.2 (1.8, 2.6)	2.1 (1.7, 2.5)	.670
HDL-C (mmol/L)	1.1 (0.9, 1.2)	1.0 (0.8, 1.2)	1.0 (0.9, 1.2)	.460
Cr (μmol/L)	871.6 ± 272.89	873.6 ± 318.89	904.5 ± 566.12	.928
Urea (mmol/L)	21.1 (16.5, 26.8)	21.4 (17.4, 27.5)	20.8 (16.1, 26.8)	.480
UA (μmol/L)	346.0 (295.0, 386.0)	333.0 (291.0, 375.0)	339.0 (296.0, 382.0)	.465
K (mmol/L)	3.8 (3.4, 4.5)	4.0 (3.4, 4.4)	3.9 (3.4, 4.5)	.965
HCO₃⁻ (mmol/L)	24.7 ± 4.11	24.9 ± 4.03	24.2 ± 4.16	.407
Ca (mmol/L)	2.1 ± 0.25	2.1 ± 0.24	2.1 ± 0.25	.393
Mg (mmol/L)	0.9 ± 0.19	0.8 ± 0.15	0.8 ± 0.15	.558
P (mmol/L)	1.6 ± 0.58	1.5 ± 0.48	1.6 ± 0.62	.974
PTH (pg/mL)	226.6 (121.8, 412.2)	218.1 (99.3, 371.5)	248.2 (123.5, 474.3)	.550
BNP (pg/mL)	361.9 (120.0, 769.0)	418.4 (164.0, 765.0)	571.8 (182.1, 1143.8)	.110

Data are presented as mean ± standard deviation, median (interquartile range) or *n* (%).

### Development of the predictive nomogram

Candidate predictors encompassed demographic indices (sex, age, BMI); clinical scores (pain NRS, NRS 2002); peritoneal effluent parameters (Eff_WBC_Pre and Eff_WBC_D1–D4); hematological markers (WBC, HGB, MCV, NEUT); inflammatory indicators (CRP, PCT); biochemical profiles (ALB, GLU, TG, TC, LDL-C, HDL-C, Cr, urea, UA); electrolytes (K, HCO₃⁻, Ca, Mg, P); and hormonal markers (PTH, BNP).

**Figure 1:**
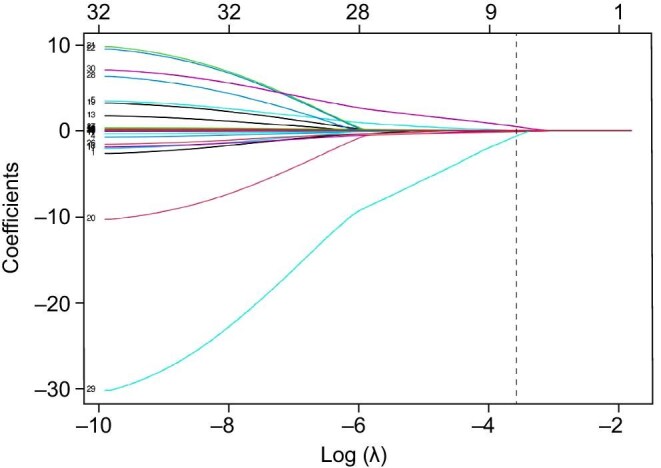
Lasso coefficient regularization paths (λ = 0.0277974026475178).

**Figure 2:**
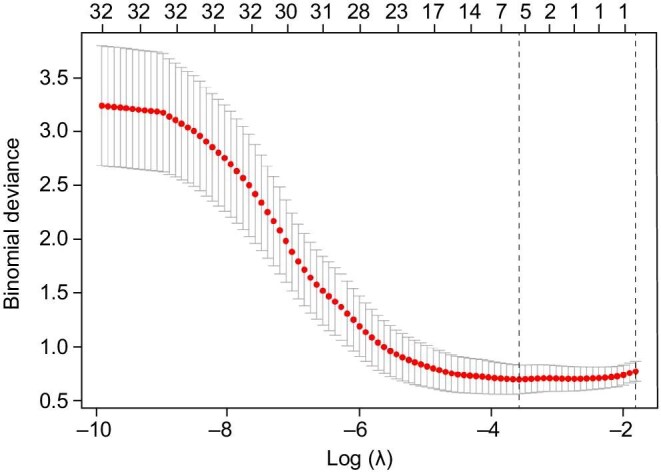
Lasso regression coefficient paths with 10-fold cross-validation (λ = 0.0277974026475178).

LASSO regression analysis applied to the training cohort refined the predictor set to five key variables. Other candidate predictors, such as NRS 2002, WBC and CRP, were not selected by the LASSO regression, which prioritizes parsimony and penalizes non-informative variables to prevent overfitting. The coefficient trajectory (Fig. 1) and cross-validation error plot (Fig. 2) demonstrated optimal model parsimony at the minimum mean squared error (MSE). The final predictors and their regression coefficients are summarized in Table 2.

**Table 2:** Coefficients from LASSO regression analysis for final predictors.

Variable	Coefficient
(Intercept)	–1.59361008
Eff_WBC_D3	0.00166687
ALB	–0.02210732
TC	–0.12339226
Mg	–0.62415380
P	0.46798058

**Figure 3:**
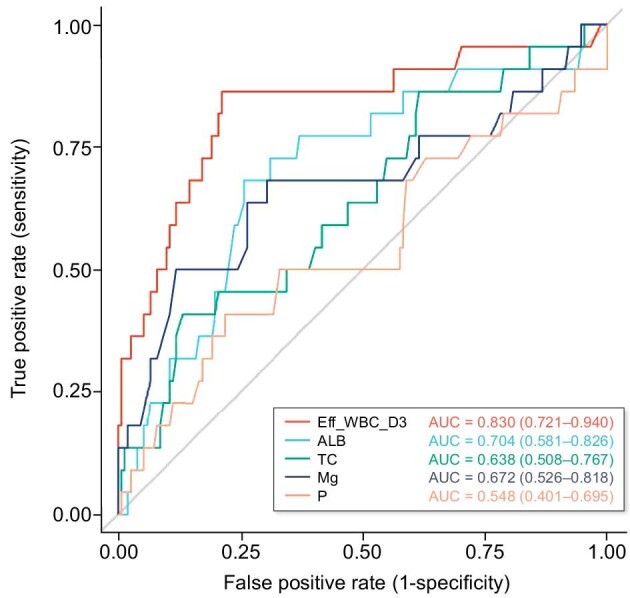
Comparison of ROC curves for individual predictive variables.

Univariate ROC analyses (Fig. 3) demonstrated discriminatory capacity of the five selected predictors, with AUC values interpreted as follows: >0.8 (excellent), >0.7 (good) and >0.5 (acceptable). Eff_WBC_D3 exhibited outstanding discrimination [AUC = 0.830, 95% confidence interval (CI) 0.721–0.940]. ALB (AUC = 0.704) and Mg (AUC = 0.672) demonstrated good to moderate discrimination, while TC showed fair predictive capacity (AUC = 0.638). Serum P (AUC = 0.548) offered limited univariate utility. These results confirm that the final model, particularly its strongest predictor (Eff_WBC_D3), possesses substantial discriminatory power for clinical use.

**Figure 4:**
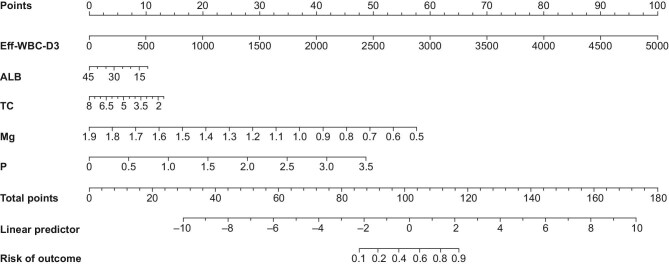
Nomogram prediction model.

Multivariate logistic regression in the training cohort (Table 3) subsequently incorporated these five independent predictors (Eff_WBC_D3, ALB, TC, P, Mg) into the final model, which was then operationalized as a clinically deployable nomogram (Fig. 4).

**Table 3:** Results of multivariate logistic regression for training cohort.

Characteristic	*N*	Event *N*	OR	95% CI	*P*-value
Eff_WBC_D3 (/100 μL)	173	22	1.32	1.15, 1.52	<.001
ALB (g/L)	173	22	0.96	0.86, 1.07	.478
TC (mmol/L)	173	22	0.75	0.39, 1.47	.406
Mg (mmol/L)	173	22	0.00	0.00, 0.55	.029
P (mmol/L)	173	22	6.97	2.05, 23.72	.002

Note: Eff_WBC_D3 was rescaled per 100 µL for regression analysis to improve interpretability.

### Model performance

**Figure 5:**
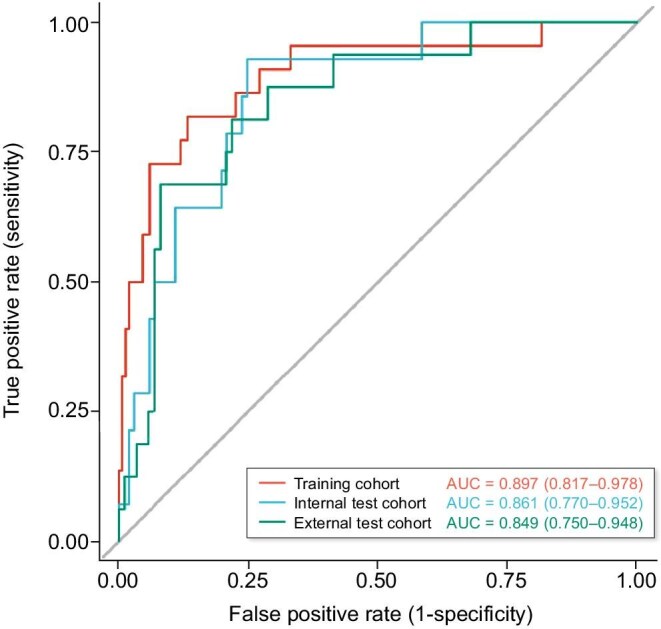
ROC curve of the prediction model in the different cohorts.

The ROC curve analysis (Fig. 5) demonstrated robust discriminative performance of the predictive model across all datasets, with AUC values consistently exceeding 0.84. The model achieved excellent discrimination in the training cohort (AUC = 0.897, 95% CI 0.817–0.978) and the internal validation cohort (AUC = 0.861, 95% CI 0.770–0.952), with a minimal optimism bias (ΔAUC = 0.036). Notably, the external validation cohort maintained a high AUC of 0.849 (95% CI 0.750–0.948), further confirming the model’s generalizability. The overlapping CIs and narrow widths (training width = 0.161; internal validation width = 0.182) demonstrate consistent predictive accuracy and high precision when applied to independent populations.

**Figure 6:**
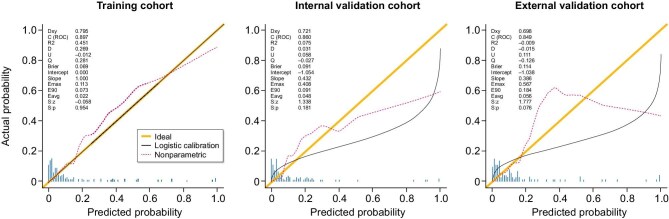
Calibration curves of the nomogram prediction model for the training, internal validation and external validation cohorts.

The calibration plots of the nomogram for the different cohorts are shown in Fig. 6 and demonstrate good agreement between the observed and predicted probabilities of treatment failure. The results indicate that the nomogram retained its validity in both the internal and external validation cohorts, with calibration curves closely approximating the ideal diagonal line, suggesting that the predicted probabilities were consistent with the actual clinical outcomes.

**Figure 7:**
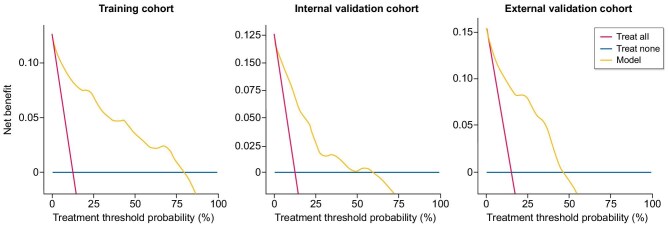
DCA of the nomogram prediction model for the training, internal validation and external validation cohorts.

The decision curve analysis (DCA; Fig. 7) further demonstrates the clinical utility of the nomogram. The DCA curves indicate that the model consistently provides a higher net benefit than either the treat-all or treat-none strategies across a broad range of threshold probabilities, supporting its substantial value in guiding individualized treatment decisions in routine clinical practice.

## DISCUSSION

This multicenter study developed and validated a novel nomogram for predicting treatment failure in CNPDP. The model, incorporating five routinely available variables, demonstrated robust performance and generalizability across independent cohorts. Its integration of a dynamic marker of treatment response (Eff_WBC_D3) with baseline nutritional and metabolic profiles provides a practical tool for early risk stratification at the bedside, addressing a critical unmet need in the management of this challenging condition.

Unlike previous models that primarily targeted culture-positive peritonitis or overall PDAP outcomes, our nomogram is uniquely tailored to CNPDP. The inclusion of Eff_WBC_D3 is a key innovation, moving beyond static baseline assessments to offer clinicians an early, dynamic readout of therapeutic efficacy during the critical first few days of empirical treatment.

Our analysis identified several predictors significantly associated with treatment failure in CNPDP patients.

Eff_WBC_D3 showed a clear positive correlation with treatment failure, consistent with previous studies [16, 17] that have highlighted persistent peritoneal inflammation as a marker of suboptimal response to empirical therapy in PDAP patients.

Both ALB and TC, which reflect patients’ nutritional and metabolic status (AUCs 0.704 and 0.638, respectively), emerged as significant predictors. Hypoalbuminemia is widely recognized as a marker of protein-energy wasting and systemic inflammation [18–20], while lower TC levels may indicate malnutrition– inflammation complex syndrome, which is prevalent in dialysis populations [21]. These findings underscore the importance of comprehensive nutritional assessment and timely intervention following peritonitis episodes.

Mg was inversely associated with treatment failure. This relationship may be explained by the pro-inflammatory effects of hypomagnesemia: (i) reduced Mg levels are directly associated with increased systemic inflammation (e.g. interleukin-6, β = –0.21) [22]; and (ii) in PD patients chronically exposed to high-glucose dialysate, low Mg exacerbates oxidative stress by impairing antioxidant defenses, which in turn amplifies inflammation-mediated vascular injury [23]. Collectively, these pathways may increase the risk of adverse clinical outcomes.

The positive association of serum phosphate with treatment failure is multifactorial. Directly, hyperphosphatemia promotes vascular inflammation and calcification, compromising host defense [24, 25]; Secondary hyperparathyroidism resulting from dysregulated calcium–phosphorus homeostasis, which may increase susceptibility to peritonitis [26]. Indirectly, the clinical management of hyperphosphatemia often necessitates dietary protein restriction, which can inadvertently exacerbate protein-energy wasting and immunodeficiency, creating a vicious cycle that hinders recovery from peritonitis [27].

The model’s strong discriminative power, together with the minimal performance decrement across the training, internal and external validation cohorts (ΔAUC = 0.036 between training and internal validation; ΔAUC = 0.048 between training and external validation), suggests promising generalizability despite the inherent heterogeneity of dialysis populations. The relatively narrow CIs in all three cohorts further support the precision and reliability of the estimates, outperforming many existing risk prediction tools in nephrology. This robust external validation provides additional evidence for the model’s applicability and stability in diverse clinical settings, supporting its potential for broader implementation in routine PD management.

Several models have been developed to predict outcomes in PDAP [10, 11, 28]. However, models such as those by Mao *et al*. (targeting general PDAP treatment failure) and Yan *et al*. (for refractory peritonitis) were derived from cohorts that included culture-positive cases [10, 11]. In contrast, our nomogram is uniquely tailored to the CNPDP subgroup, which faces distinct clinical challenges due to the absence of microbiological guidance. This specific focus, combined with the integration of a dynamic marker of treatment response (Eff_WBC_D3), constitutes a key advancement over previous static models. Our approach provides clinicians with early, actionable risk information during the critical phase of empirical therapy. Furthermore, the model achieves robust discriminative performance (AUCs 0.897–0.849 across cohorts), which compares favorably to the typical 0.70–0.79 range of conventional scores [11, 25], while utilizing only five routinely available variables. This parsimonious design enhances its practicality and facilitates real-world implementation at the bedside.

To enhance clinical accessibility, the developed nomogram has been deployed as an interactive web-based tool (https://liuliangmianhua.shinyapps.io/dynnomapp/), allowing real-time, point-of-care risk calculation to support individualized management of CNPDP patients. A screenshot of the web interface is provided in the Supplementary data, Fig. S2, to illustrate its functionality. Notably, the inverse association identified for serum magnesium highlights a potentially modifiable risk factor that merits further prospective interventional research. Overall, this easily accessible tool has the potential to facilitate personalized decision-making, enable timely treatment adjustments, and optimize resource allocation in the clinical management of CNPDP.

### Limitations

Several limitations should be acknowledged.

First, the retrospective design carries an inherent risk of residual confounding from unmeasured variables, such as genetic predispositions or socioeconomic factors, despite rigorous statistical adjustment.

Second, the model’s performance in specific subgroups (e.g. elderly or highly comorbid patients) could not be fully assessed due to limited sample size in these subsets.

Third, the model does not incorporate longitudinal biomarker trends (e.g. Eff_WBC trajectory slopes), which may limit its ability to capture subtle dynamic changes relevant to treatment response.

Fourth, while the online tool is convenient, its implementation may face challenges in resource-limited settings with poor internet access; future work could develop paper-based versions or electronic health record integrations.

Finally, while the model was validated in independent Asian cohorts, its generalizability to non-Asian populations and diverse healthcare settings requires further multinational prospective validation.

Despite these limitations, this rigorously validated model provides a solid foundation for clinical implementation, prospective evaluation and continued optimization in the management of CNPDP.

### Conclusions

In conclusion, this rigorously validated predictive model provides clinicians with a robust, practical tool for individualized risk assessment of treatment failure in CNPDP patients. Its strong discriminative performance, clear clinical interpretability and streamlined structure enable timely, targeted interventions and more efficient resource allocation. The resulting nomogram offers a readily deployable bedside solution to support personalized management for this challenging population. Future studies should validate its performance in larger, more diverse cohorts and investigate the additional value of dynamic biomarker monitoring and targeted interventions to further improve outcomes.

## Supplementary Material

sfaf390_Supplemental_Files

## Data Availability

The datasets generated during and/or analyzed during the current study are available from the corresponding author on reasonable request. Contact: Xiang Li, E-mail: jyfylx@163.com.
