# Supplementary material for: Development and validation of a nomogram for predicting treatment failure in culture-negative peritoneal dialysis–associated peritonitis
Source: Clin Kidney J. 2025 Dec 13;19(2):sfaf390. doi: 10.1093/ckj/sfaf390 (PMC12862217; doi:10.1093/ckj/sfaf390)

Fig.S1 Flowchart of patient selection
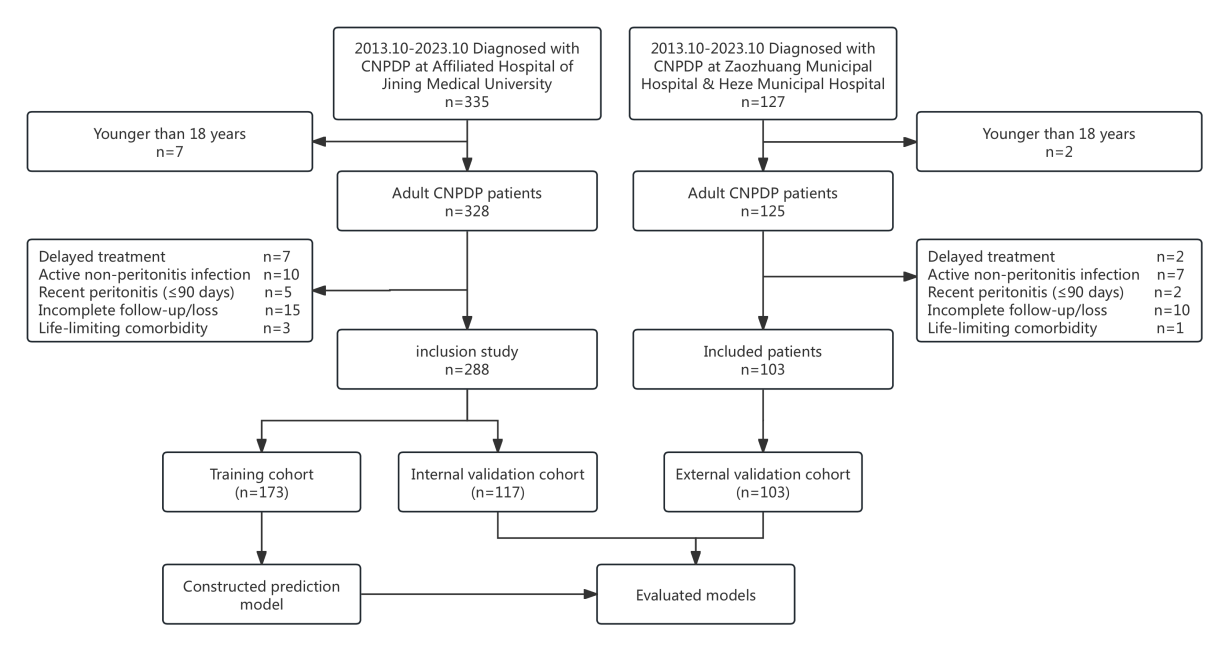


Fig.S2 Screenshot of the interactive web-based nomogram tool. The online tool requires inputs for PD effluent WBC after 3 days of therapy, albumin (ALB), total cholesterol (TC), magnesium (Mg), and phosphorus (P). The displayed example, with all parameters set, predicts a treatment failure probability of 76.5% (95% CI: 47% - 92.3%), as shown in the 'Numerical Summary' panel.


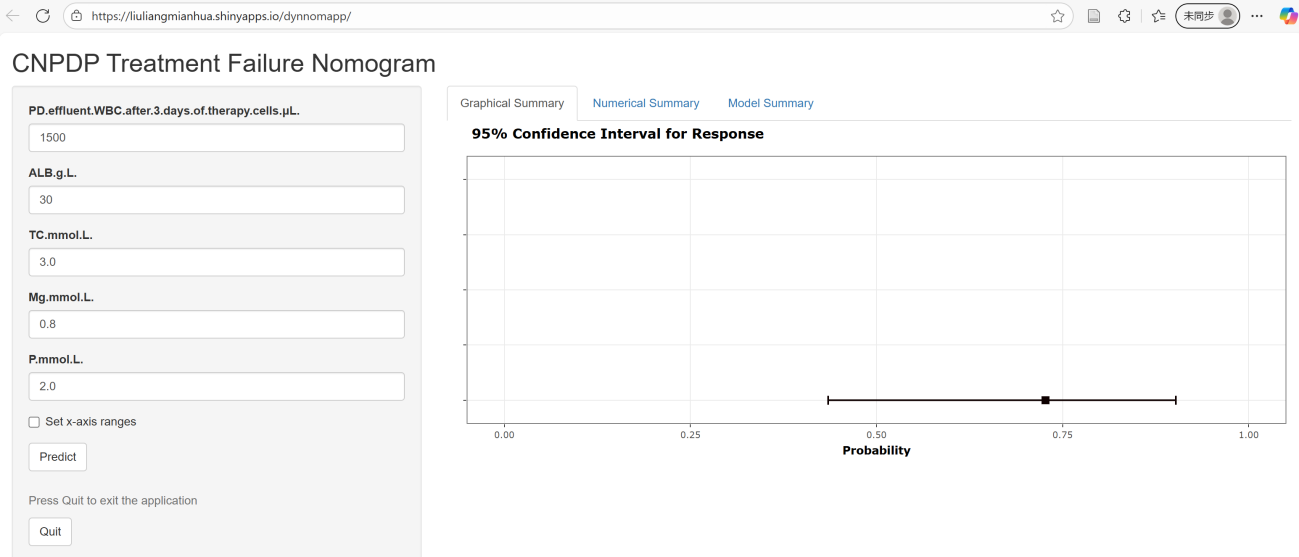

Supplement: sfaf390_Supplemental_Files [file sfaf390_supplemental_files.zip › 1217 supplementary material.docx]
