# Supplementary figures and images for: Development and validation of a nomogram for predicting treatment failure in culture-negative peritoneal dialysis–associated peritonitis
Source: Clin Kidney J. 2025 Dec 13;19(2):sfaf390. doi: 10.1093/ckj/sfaf390 (PMC12862217; doi:10.1093/ckj/sfaf390)

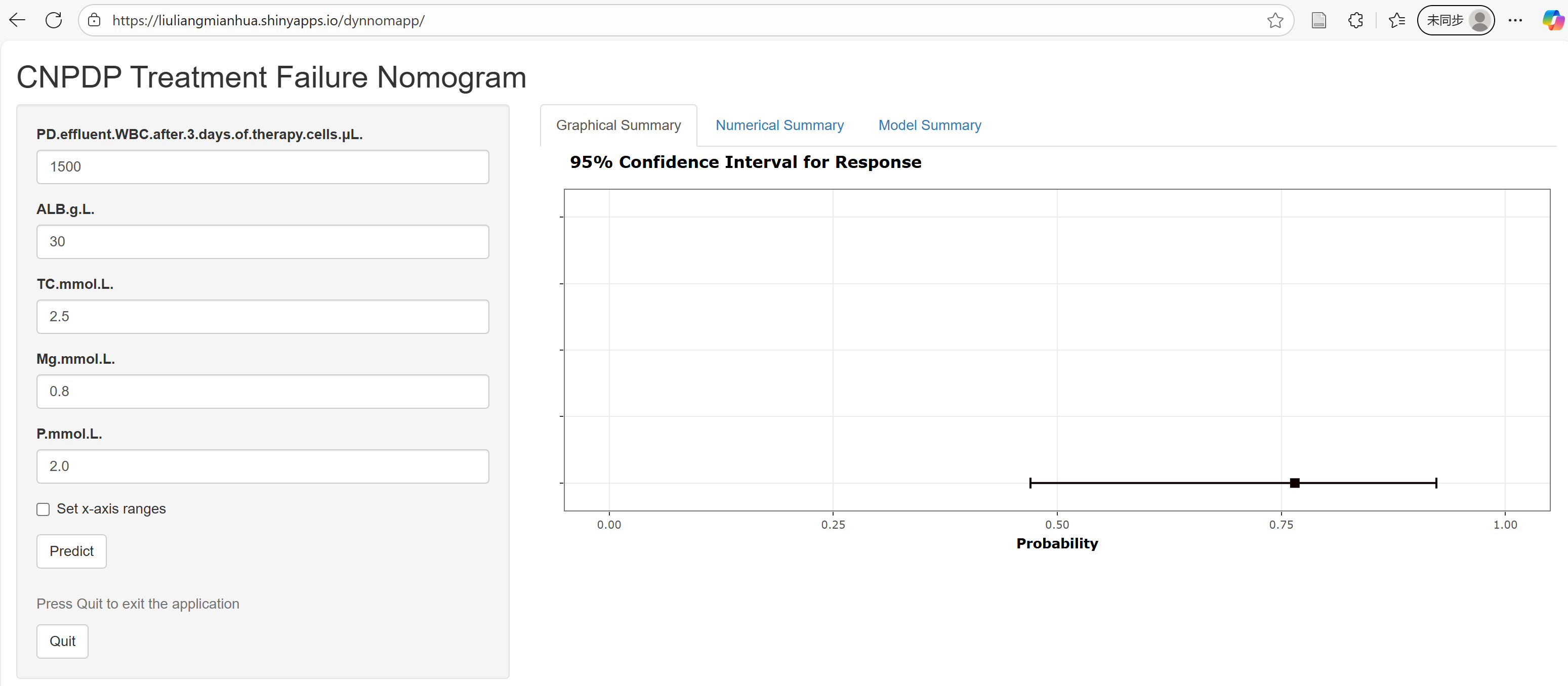

Supplement: sfaf390_Supplemental_Files [file sfaf390_supplemental_files.zip › Fig.S2 Screenshot of the interactive web-based nomogram tool.png]
